# The role of carbon dioxide in nematode behaviour and physiology

**DOI:** 10.1017/S0031182019001422

**Published:** 2020-07

**Authors:** Navonil Banerjee, Elissa A. Hallem

**Affiliations:** Department of Microbiology, Immunology, and Molecular Genetics, University of California, Los Angeles, CA, USA

**Keywords:** Carbon dioxide, chemotaxis, *C. elegans*, hookworms, nematodes, parasitic nematodes, sensory behaviour, *Strongyloides*

## Abstract

Carbon dioxide (CO_2_) is an important sensory cue for many animals, including both parasitic and free-living nematodes. Many nematodes show context-dependent, experience-dependent and/or life-stage-dependent behavioural responses to CO_2_, suggesting that CO_2_ plays crucial roles throughout the nematode life cycle in multiple ethological contexts. Nematodes also show a wide range of physiological responses to CO_2_. Here, we review the diverse responses of parasitic and free-living nematodes to CO_2_. We also discuss the molecular, cellular and neural circuit mechanisms that mediate CO_2_ detection in nematodes, and that drive context-dependent and experience-dependent responses of nematodes to CO_2_.

## Introduction

Carbon dioxide (CO_2_) is an important sensory cue for animals across diverse phyla, including Nematoda (Lahiri and Forster, 2003; Shusterman and Avila, 2003; Bensafi *et al*., 2007; Smallegange *et al*., 2011; Carrillo and Hallem, 2015). While the CO_2_ concentration in ambient air is approximately 0.038% (Scott, 2011), many nematodes encounter much higher levels of CO_2_ in their microenvironment during the course of their life cycles. For instance, parasitic nematodes may encounter high CO_2_ concentrations released from potential hosts as a byproduct of respiration or from the host feces within which they develop (Byrnes *et al*., 1997; Buszewski *et al*., 2007; Carrillo and Hallem, 2015). CO_2_ concentrations are also high in specific tissues such as the venous bloodstream, lungs and intestine (Jensen and Jorgensen, 1994; Rotbart *et al*., 2017), suggesting CO_2_ may be an important intra-host cue for parasitic nematodes (Hawdon and Schad, 1990; Bekelaar *et al*., 2018, 2019). Moreover, many free-living nematodes are found in rotting vegetation, where CO_2_ levels are often high (Burg and Burg, 1965; Felix and Duveau, 2012). Therefore, nematodes must detect and respond appropriately to elevated CO_2_ concentrations to survive, navigate through their microenvironment and propagate.

CO_2_ may serve as a beneficial or detrimental cue for nematodes depending on specific circumstances (Carrillo and Hallem, 2015). For instance, in the case of parasitic nematodes, CO_2_ may be necessary to promote parasite–host interactions and thus support their parasitic life cycle. For free-living nematodes, high CO_2_ levels present in their natural habitats may act as signals for food, predators, pathogens or conspecifics (Carrillo and Hallem, 2015). Because CO_2_ is a complex cue that can have either a positive or negative valence, it is not surprising that different species of nematodes have developed distinct behavioural and physiological responses to CO_2_. Moreover, many species, both free-living and parasitic, exhibit CO_2_ responses that vary with context, previous experience and/or life stage. Recent studies of the free-living model nematode *Caenorhabditis elegans* have provided insight into the cellular and molecular mechanisms that drive and modulate CO_2_-evoked responses. In contrast, the mechanisms that promote the diverse responses of parasitic nematodes to CO_2_ have not yet been elucidated due to the historic lack of tools required for molecular genetic studies of these worms. However, as a result of recent developments in molecular genetic techniques, we are now in a position to interrogate the neural circuits and molecular signals that promote CO_2_ responses in parasitic nematodes. The findings from these studies will enhance our understanding of the role of CO_2_ in sculpting parasite–host interactions and may enable the development of novel strategies to combat harmful nematode infections. Here, we review our existing knowledge of how various nematode species respond behaviourally and physiologically to CO_2_. We also discuss how CO_2_ responsiveness can be modulated based on context, previous experience and life stage.

## Responses of mammalian-parasitic nematodes to CO_2_

### Introduction to parasitic nematodes of mammals

Mammalian-parasitic nematodes infect over a billion people worldwide and are a major cause of morbidity in low-resource areas (Boatin *et al*., 2012). Infections with soil-transmitted nematodes can cause chronic gastrointestinal distress, stunted growth and cognitive impairment in children, anaemia and even fatality in infants and immunocompromised individuals (Lustigman *et al*., 2012). Infections with vector-transmitted nematodes can cause severe symptoms such as permanent disfigurement and blindness (Lustigman *et al*., 2012). Additionally, parasitic nematodes that infect livestock are an enormous source of economic burden (Jasmer *et al*., 2003). Current treatments for infections depend on anthelminthic drugs that reduce the worm burden in heavier infections but do not prevent reinfections, with the result that reinfection is common in endemic areas (Prichard *et al*., 2012). In addition, drug resistance resulting from mass drug administration is a major challenge for the treatment of nematode-infected livestock (Kumar *et al*., 2013; Roeber *et al*., 2013; Emery *et al*., 2016; Learmount *et al*., 2016) and is expected to be a concern for the treatment of nematode-infected humans in the near future (Keiser and Utzinger, 2008; Diawara *et al*., 2013; Repetto *et al*., 2018). The drugs currently available are also not sufficient to eliminate human infections in all cases, at least by following the administration schedules under practice (Repetto *et al*., 2018).

Many of these nematodes are gastrointestinal parasites with a developmentally arrested infective larval stage that inhabits the environment and infects hosts following either skin penetration or passive ingestion, depending on the species (Gang and Hallem, 2016; Bryant and Hallem, 2018). The infective larval stages of these species respond robustly to a diverse array of host and environmental sensory cues, including CO_2_ (Gang and Hallem, 2016; Bryant and Hallem, 2018). In addition, many parasitic nematodes may rely on sensory cues inside the host body, including CO_2_, to re-initiate development upon host entry, direct somatic migration and establish a successful infection (Hawdon and Schad, 1990, 1992; Hawdon *et al*., 1992; Bekelaar *et al*., 2018, 2019).

### Responses of skin-penetrating nematodes to CO_2_

Skin-penetrating nematodes such as the human-parasitic hookworms *Ancylostoma duodenale* and *Necator americanus* and the human-parasitic threadworm *Strongyloides stercoralis* are gastrointestinal parasites that infect hosts as developmentally arrested third-stage larvae (iL3s) (Roberts *et al*., 2005; Nutman, 2017; Velikkakam *et al*., 2017). The iL3s are soil-dwelling and actively engage in host seeking using a variety of host-associated sensory cues (Gang and Hallem, 2016; Bryant and Hallem, 2018). These parasites generally have narrow host ranges, infecting only a limited number of host species (Haley, 1961; Bezubik, 1965; Nolan *et al*., 2007; Viney and Lok, 2007; Viney and Kikuchi, 2017). After invading a host by skin penetration, the iL3s resume development inside the host, a process called activation (Stoltzfus *et al*., 2012, 2014). The nematodes then migrate through the host body to their final destination, the small intestine, where they reside as parasitic adults (Roberts *et al*., 2005; Nutman, 2017; Velikkakam *et al*., 2017). The adults reproduce in the small intestine, and then the eggs or young larvae, depending on the species, exit the host body in feces. The nematodes inhabit the feces until they develop into iL3s (Roberts *et al*., 2005; Nutman, 2017; Velikkakam *et al*., 2017). In most species, all of the progeny of the parasitic adults develop directly into iL3s. However, *Strongyloides* species can cycle through one or a limited number of free-living generations on the feces before developmentally arresting as iL3s (Roberts *et al*., 2005). *Strongyloides stercoralis* can also pass through multiple generations inside the same host through autoinfective cycles (Roberts *et al*., 2005).

Many skin-penetrating nematodes show behavioural responses to CO_2_. For example, iL3s of the dog hookworm *Ancylostoma caninum* display increased nictation in the presence of CO_2_ (Granzer and Haas, 1991). Nictation is a specialized behaviour displayed by many parasitic nematodes in which the worm stands on its tail and waves its head in the air to facilitate attachment to mobile hosts (Granzer and Haas, 1991; Bryant and Hallem, 2018). In addition, both *Ancylostoma caninum* and *Strongyloides stercoralis* iL3s exhibit increased movement when exposed to human breath, and this behaviour is not observed when CO_2_ is removed from the breath (Sciacca *et al*., 2002). Similarly, the human-parasitic hookworms *Ancylostoma duodenale* and *Necator americanus* display increased activity in response to CO_2_ in combination with heat and/or moisture (Haas *et al*., 2005). The similar responses of *Strongyloides stercoralis* and hookworms to CO_2_ is particularly notable given their phylogenetic divergence, with *Strongyloides stercoralis* in clade IV and hookworms in clade V (Blaxter and Koutsovoulos, 2015; Blaxter *et al*., 2016). However, these studies did not look at migration in CO_2_ gradients, and whether CO_2_ was an attractant or repellent was not clear.

**Fig. 1.**
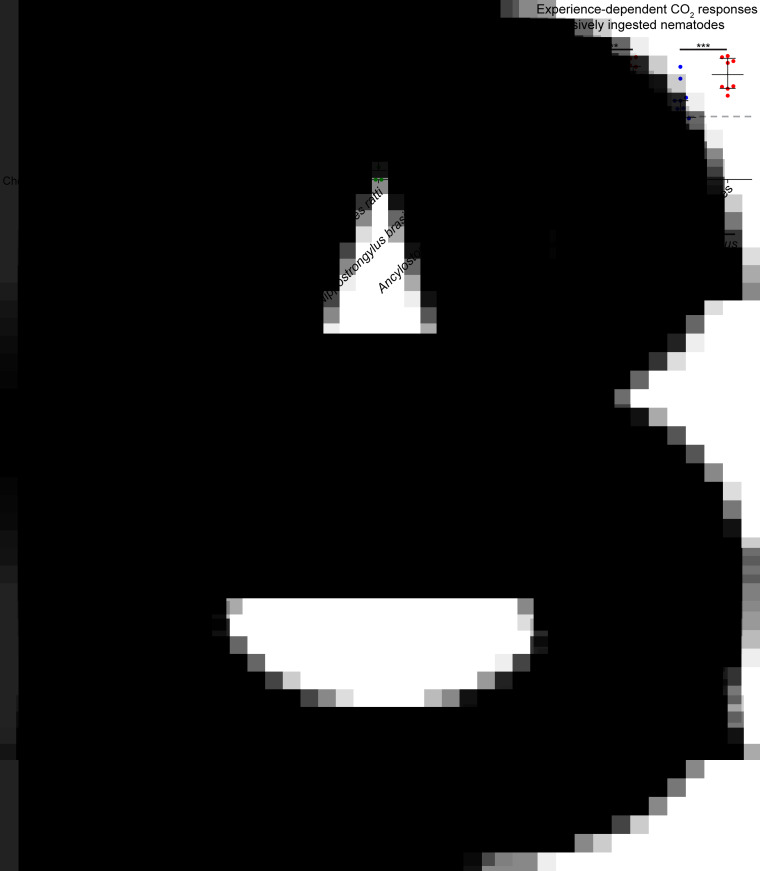
Behavioural responses of mammalian-parasitic nematodes to CO_2_. (A) A CO_2_-chemotaxis assay. CO_2_ and air are pumped into opposite sides of a 10 cm plate. Infective larvae (iL3s) are placed at the centre and allowed to migrate for 1 h. A chemotaxis index is then calculated according to the formula indicated; a positive index indicates attraction and a negative index indicates repulsion. (B) Behavioural responses of skin-penetrating iL3s to 10% CO_2_. All species tested are repelled by CO_2_. Data are from Castelletto *et al*. (2014) and Ruiz *et al*. (2017). (C) Experience-dependent changes in CO_2_ responsiveness in the passively ingested nematodes *Heligmosomoides polygyrus* and *Haemonchus contortus*. CO_2_ responses switch from repulsion (in *Heligmosomoides polygyrus*) or neutral (in *Haemonchus contortus*) to attraction following removal from host feces for days to weeks. *Heligmosomoides polygyrus* was tested with 10% CO_2_; *Haemonchus contortus* was tested with 15% CO_2_. Figure adapted from Ruiz *et al*. (2017). Graphs show medians and interquartile ranges. ****P < 0.0001, ***P < 0.001, Mann–Whitney test for each species.

More recent studies demonstrated that skin-penetrating iL3s of the human parasites *Strongyloides stercoralis* and *Ancylostoma ceylanicum* and the rat parasites *Strongyloides ratti* and *Nippostrongylus brasiliensis* are repelled by CO_2_ in CO_2_-chemotaxis assays (Fig. 1A, B) (Castelletto *et al*., 2014; Ruiz *et al*., 2017). A lack of attraction towards CO_2_ is consistent with the route of infection of skin-penetrating nematodes, since mammalian skin surfaces emit low concentrations of CO_2_ (Alkalay *et al*., 1971). On the other hand, fecal deposits contain high levels of CO_2_ resulting from aerobic respiration of fecal bacteria (Jensen and Jorgensen, 1994; de Lacy Costello *et al*., 2014; Rotbart *et al*., 2017), and CO_2_ repulsion may drive these iL3s off of host feces and into the environment in search of new hosts.

### Responses of passively ingested nematodes to CO_2_

Many passively ingested gastrointestinal nematodes have a motile environmental iL3 stage that invades hosts after being swallowed. For example, iL3s of the ruminant parasite *Haemonchus contortus* inhabit the soil and infect after being swallowed by grazing animals (O'Connor *et al*., 2006). After entering a host, the nematodes exsheath in the rumen and travel to the abomasum, where they develop into parasitic adults (Laing *et al*., 2013). Similarly, the murine gastrointestinal parasite *Heligmosomoides polygyrus* has an iL3 stage that can infect mice either from feces during coprophagy or from the fur during grooming (Hernandez and Sukhdeo, 1995). Despite their passive route of infection, both *Haemonchus contortus* and *Heligmosomoides polygyrus* actively migrate towards host-associated sensory cues. This suggests that these species use host-associated cues to position themselves in the vicinity of potential hosts, where they are more likely to be ingested (Hernandez and Sukhdeo, 1995; Castelletto *et al*., 2014; Ruiz *et al*., 2017; Bryant *et al*., 2018).

Examination of the CO_2_-evoked behaviours of *Haemonchus contortus* and *Heligmosomoides polygyrus* revealed that both species show experience-dependent responses to CO_2_ (Fig. 1C) (Castelletto *et al*., 2014; Ruiz *et al*., 2017). In the case of *Heligmosomoides polygyrus*, iL3s extracted directly from feces are repelled by CO_2_, while iL3s that have been removed from feces for multiple days – a condition designed to mimic the soil environment of iL3s – are attracted to CO_2_ (Ruiz *et al*., 2017). This shift in CO_2_ preference appears to occur as a result of the drop in ambient CO_2_ levels experienced by the iL3s after they migrate off feces, since cultivating iL3s off feces under high CO_2_ conditions (2.5% CO_2_) prevents the behavioural switch. The initial repulsion from CO_2_ experienced by *Heligmosomoides polygyrus* iL3s on feces may enable them to disperse off of feces and into the environment to host seek. Following a prolonged period without feces, CO_2_ attraction may drive them towards new hosts or fresh host feces to increase their chances of host entry through ingestion (Ruiz *et al*., 2017).

In the case of *Haemonchus contortus*, iL3s directly removed from feces are neutral to CO_2_, whereas iL3s that have been removed from feces for a week or more are attracted to CO_2_ (Castelletto *et al*., 2014; Ruiz *et al*., 2017). This experience-dependent shift in CO_2_-evoked behaviour may enable the iL3s to migrate towards the mouths of grazing ruminants, whose breath emits high concentrations of CO_2_ (Ruiz *et al*., 2017). In contrast to *Heligmosomoides polygyrus* and *Haemonchus contortus*, the skin-penetrating nematodes *Ancylostoma ceylanicum*, *Strongyloides stercoralis* and *Strongyloides ratti* do not display this flexibility in their behavioural responses to CO_2_. Thus, experience-dependent plasticity towards CO_2_ may be unique to passively ingested nematodes (Ruiz *et al*., 2017). However, skin-penetrating nematodes do show other forms of sensory plasticity, including experience-dependent thermal plasticity and temperature-dependent olfactory plasticity (Lee *et al*., 2016; Bryant *et al*., 2018). This suggests that experience-dependent responses to CO_2_ may not be beneficial for skin-penetrating nematodes, likely because the skin surface of mammals emits only very low levels of CO_2_ (Alkalay *et al*., 1971).

### The role of CO_2_ in mammalian-parasitic nematode development and physiology

In addition to being a robust behavioural cue for parasitic nematodes, CO_2_ is also an important regulator of their development and physiology. For example, CO_2_ stimulates exsheathment and activation (exit from the developmentally arrested iL3 stage) in passively ingested ruminant parasites such as *Haemonchus contortus* (Rogers and Sommerville, 1960; Taylor and Whitlock, 1960; Sommerville, 1964; Bekelaar *et al*., 2018, 2019). However, the requirement for CO_2_ during exsheathment varies across species. CO_2_ is an absolute requirement for the exsheathment of *Haemonchus contortus* iL3s, whereas CO_2_ enhances but is not required for exsheathment in other passively ingested abomasal nematodes (Bekelaar *et al*., 2018). In the dog hookworm *Ancylostoma caninum*, CO_2_ is not required for activation but results in a slight increase in the rate of activation (Hawdon and Schad, 1990).

The role of CO_2_ is not limited to exsheathment and activation. CO_2_, in combination with O_2_, also regulates the development of *Strongyloides ratti* into either free-living adults or iL3s (Taylor and Weinstein, 1990). In addition, CO_2_ stimulates egg hatching in the giant roundworm *Ascaris lumbricoides*, a human-parasitic species that infects when eggs containing developmentally arrested infective larvae are swallowed by hosts as a result of fecal–oral contamination (Fairbairn, 1961; Dold and Holland, 2011). Finally, CO_2_ is required for the *in vitro* development of parasitic larvae in the pig roundworm *Ascaris suum* (Douvres and Urban, 1983). Thus, CO_2_ influences both behaviour and development in many if not all mammalian-parasitic nematode species.

## Responses of entomopathogenic nematodes to CO_2_

### Introduction to entomopathogenic nematodes

Entomopathogenic nematodes (EPNs) are parasites that infect and kill insects (Dillman and Sternberg, 2012). They are considered beneficial for humans due to their role as biological agents for pest control, and are likely also important for maintaining balanced ecosystems in nature. EPNs of the genera *Heterorhabditis* and *Steinernema* have been successfully employed commercially against insect agricultural pests (Liu *et al*., 2000; Grewal *et al*., 2005; Dillman and Sternberg, 2012; Labaude and Griffin, 2018). The geographical distribution of EPNs spans all continents except Antarctica (Hominick, 2002). Some EPNs, such as *Steinernema carpocapsae* and *Heterorhabditis bacteriophora*, are generalists that can infect many different insects; in contrast, other EPNs have very narrow host ranges (Peters, 1996). For example, the specialist *Steinernema scapterisci* specifically infects mole crickets, and the specialist *Steinernema diaprepesi* specifically infects the larval stages of the citrus pest *Diaprepes abbreviatus* (Nguyen and Smart, 1991; Nguyen and Hunt, 2007; Ali *et al*., 2010). EPNs infect only as third-stage larvae called infective juveniles (IJs); the IJ stage of EPNs is equivalent to the iL3 stage of mammalian-parasitic nematodes (Dillman *et al*., 2012*a*). IJs enter their insect hosts through a body orifice such as the mouth, spiracles or anus; IJs of some species can also penetrate directly through the cuticle (Bedding and Molyneux, 1982; Kaya and Gaugler, 1993). The IJs then enter the insect haemocoel and release a bacterial symbiont from their intestine (Bedding and Molyneux, 1982; Kaya and Gaugler, 1993). Toxins secreted by the nematode and the bacteria kill the insect, typically within 48 h (Kaya and Gaugler, 1993; Lu *et al*., 2017; Chang *et al*., 2019). The nematodes then feed on the insect cadaver and complete their parasitic life cycle. The nematodes can cycle through multiple generations in the host cadaver until resources are depleted, at which point new IJs form and disperse into the environment to seek out new hosts (Kaya and Gaugler, 1993).

### The role of CO_2_ in the host-seeking behaviours of EPNs

The host-seeking strategies of EPNs vary across species. Some species are considered ‘cruisers’ that actively migrate towards stationary hosts, other species are considered ‘ambushers’ that remain relatively stationary and nictate to facilitate attachment to mobile hosts, and still other species use an intermediate strategy (Campbell and Gauger, 1993; Lewis, 2002; Lewis *et al*., 2006). However, both ambushers and cruisers are capable of migrating towards host-emitted chemosensory cues, suggesting that all EPNs engage in chemosensory-driven navigation towards hosts (Schmidt and All, 1979; Pye and Burman, 1981; O'Halloran and Burnell, 2003; Hallem *et al*., 2011*a*; Dillman *et al*., 2012*b*; Castelletto *et al*., 2014; Lee *et al*., 2016). Some EPNs in the genus *Steinernema* also engage in a unique jumping behaviour where the IJ stands on its tail and then propels itself into the air, presumably to facilitate host attachment as well as transport to new niches (Campbell and Kaya, 1999). Jumping can be stimulated by exposure to host-emitted chemosensory cues (Campbell and Kaya, 1999, 2000; Hallem *et al*., 2011*a*; Dillman *et al*., 2012*b*).

**Fig. 2.**
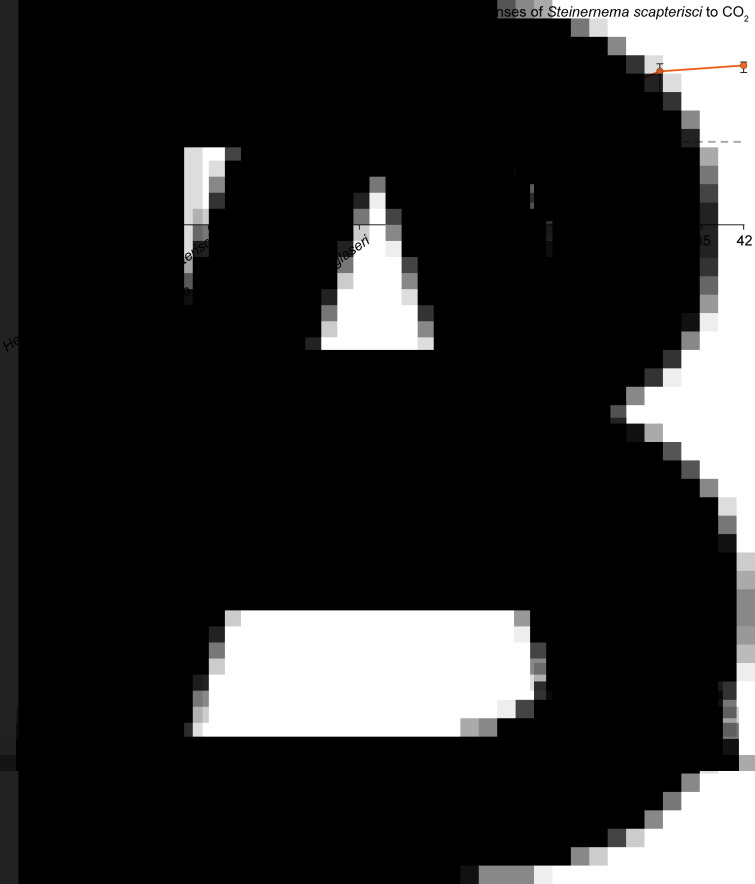
Behavioural responses of entomopathogenic nematodes (EPNs) to CO_2_. (A) Behavioural responses of the infective juveniles (IJs) of various EPN species to CO_2_ in a chemotaxis assay (Fig. 1A). All EPNs tested showed attraction to 2.5% CO_2_. Data are from Dillman *et al*. (2012*b*) and Hallem *et al*. (2011*a*). (B) Age-dependent changes in the CO_2_ preferences of *Steinernema scapterisci* IJs. IJs were grown at room temperature (approximately 22 °C) and then incubated at 15 °C until the indicated time points (days post-collection). CO_2_ responsiveness changes from repulsion to attraction with age. IJs were tested with 1% CO_2_. Data are from Lee *et al*. (2016). The graph shows medians and interquartile ranges.

Many EPNs, including *Heterorhabitis bacteriophora*, *Steinernema carpocapsae*, *Steinernema riobrave*, *Steinernema scapterisci* and *Steinernema glaseri*, are attracted to CO_2_ (Fig. 2A) (Gaugler *et al*., 1980, 1991; Lewis *et al*., 1993; Robinson, 1995; Hallem *et al*., 2011*a*; Dillman *et al*., 2012*b*; Lee *et al*., 2016). This group includes both specialists and generalists, and both ambushers and cruisers. In addition, CO_2_ stimulates jumping in *Steinernema carpocapsae*, *Steinernema riobrave* and *Steinernema scapterisci* IJs at concentrations as low as 0.08% (approximately two times higher than atmospheric levels), suggesting that jumping is highly sensitive to environmental CO_2_ (Hallem *et al*., 2011*a*; Dillman *et al*., 2012*b*). Both the attractive responses of EPNs towards the odour of live insect hosts and jumping responses to host odour are decreased when CO_2_ is chemically removed using a soda lime filter, illustrating the importance of CO_2_ for host seeking (Gaugler *et al*., 1991; Dillman *et al*., 2012*b*). However, the extent to which host attraction is reduced in the absence of CO_2_ varies across different EPN–host combinations (Dillman *et al*., 2012*b*). Thus, EPNs use CO_2_ in combination with host-specific olfactory cues to migrate towards insects. CO_2_ also acts synergistically with plant root volatiles to attract some EPNs to plants infested with insects (Turlings *et al*., 2012).

Like some mammalian-parasitic nematodes, some EPNs exhibit plasticity in their olfactory responses to CO_2_. For example, the IJs of *Steinernema scapterisci* are repelled by CO_2_ immediately after emergence from the insect host, but their response shifts to robust attraction over the course of weeks (Fig. 2B) (Lee *et al*., 2016). In addition, the rate at which this shift occurs varies depending on the cultivation temperature of the IJs, with the shift occurring more rapidly in IJs cultured at 15 °C relative to IJs cultured at 25 °C (Lee *et al*., 2016). The change in CO_2_ preference correlates with a corresponding change in responses to host odours, including the odour of its natural host, the mole cricket (Lee *et al*., 2016). The strong repulsion of newly emerged *Steinernema scapterisci* IJs to CO_2_ and host odours may serve as a dispersal mechanism to drive them towards new niches. The mechanisms that drive the change in CO_2_ preference following host emergence in *Steinernema scapterisci* have not yet been elucidated. An intriguing possibility is that the CO_2_ repulsion exhibited by IJs immediately following host emergence could result from the elevated levels of CO_2_ experienced inside the decaying insect cadaver, similar to the way in which CO_2_ repulsion in *Heligmosomoides polygyrus* iL3s results from the elevated levels of CO_2_ experienced on mammalian feces (Ruiz *et al*., 2017). However, additional experiments will be necessary to determine whether the change in CO_2_ preference over time (or age) in *Steinernema scapterisci* is in fact regulated by ambient CO_2_ levels.

## Responses of plant-parasitic nematodes to CO_2_

### Introduction to plant-parasitic nematodes

Plant-parasitic nematodes (PPNs) are a major cause of agricultural crop damage throughout the world. It has been estimated that PPNs are responsible for approximately 100 billion dollars of crop loss per year worldwide (Jasmer *et al*., 2003; Wrather *et al*., 2003). Of over 4100 species of PPNs that have been identified (Decraemer and Hunt, 2006), the ones that cause the most severe economic loss are the nematodes that infect the roots of major agricultural crops (Bernard *et al*., 2017). These PPNs prevent water and nutrient uptake by plant roots, which results in greatly reduced crop quality and yield (Bernard *et al*., 2017).

### Responses of PPNs to CO_2_

CO_2_ is ubiquitously produced by the roots of plants. Several studies have demonstrated an important role for CO_2_ in mediating attraction of PPNs to their host plants. For example, the stem nematode *Ditylenchus dipsaci*, which infects onion and garlic, migrates towards CO_2_ (Klingler, 1972; Viglierchio, 1990). Many other PPNs, including species from the genera *Ditylenchus*, *Meloidogyne*, *Heterodera* and *Pratylenchus*, are also attracted to CO_2_ (Johnson and Viglierchio, 1961; Prot, 1980; McCallum and Dusenbery, 1992; Robinson, 1995). In the case of *Meloidogyne incognita*, attraction to tomato root volatiles appears to be due to the presence of O_2_ and CO_2_ in the volatile mix (McCallum and Dusenbery, 1992). However, a more recent study found that for *Meloidogyne hapla*, the attractant is not CO_2_ itself but rather the low pH environment created by dissolved CO_2_ (Wang *et al*., 2009). In the case of the pine wilt nematode *Bursaphelenchus xylophilus*, the fourth-stage juveniles (J_IV_s) are repelled by CO_2_. CO_2_ repulsion by J_IV_s plays an important role in dispersal from its insect vector, the pine sawyer beetle, into the pine tree (Wu *et al*., 2019). *Bursaphelenchus xylophilus* J_IV_s enter the beetle tracheal system, where they are transported by the beetle to new pine trees. As the beetle matures and feeds on the pine tree, CO_2_ levels in the beetle tracheal system increase. Once CO_2_ levels reach a certain concentration, CO_2_ repulsion drives the J_IV_s out of the beetle spiracles and into the pine tree (Wu *et al*., 2019). Thus, the responses of PPNs to CO_2_ vary greatly across species. A better understanding of how other PPNs respond to CO_2_ may enable the development of new biocontrol strategies.

## Responses of free-living nematodes to CO_2_

### Introduction to free-living nematodes

Free-living nematodes are found in a wide range of ecological habitats. These include various types of soil, sediment and organic matter, as well as marine and freshwater environments. Free-living nematodes use a wide variety of sensory cues to navigate their environment in search of food and mates, and to escape from predators and pathogens. CO_2_ is universally present in terrestrial and aquatic habitats, and may serve as an important cue for survival and propagation of these nematodes. The most well-studied free-living nematode is the model worm *Caenorhabditis elegans*. *C. elegans* is commonly found in microbe-rich environments such as those of fallen rotting fruits (Felix and Duveau, 2012), where CO_2_ is produced as one of many microbial byproducts. Consequently, *C. elegans* displays several behavioural and physiological responses to CO_2_.

### Behavioural responses of *C. elegans* to carbon dioxide

**Fig. 3.**
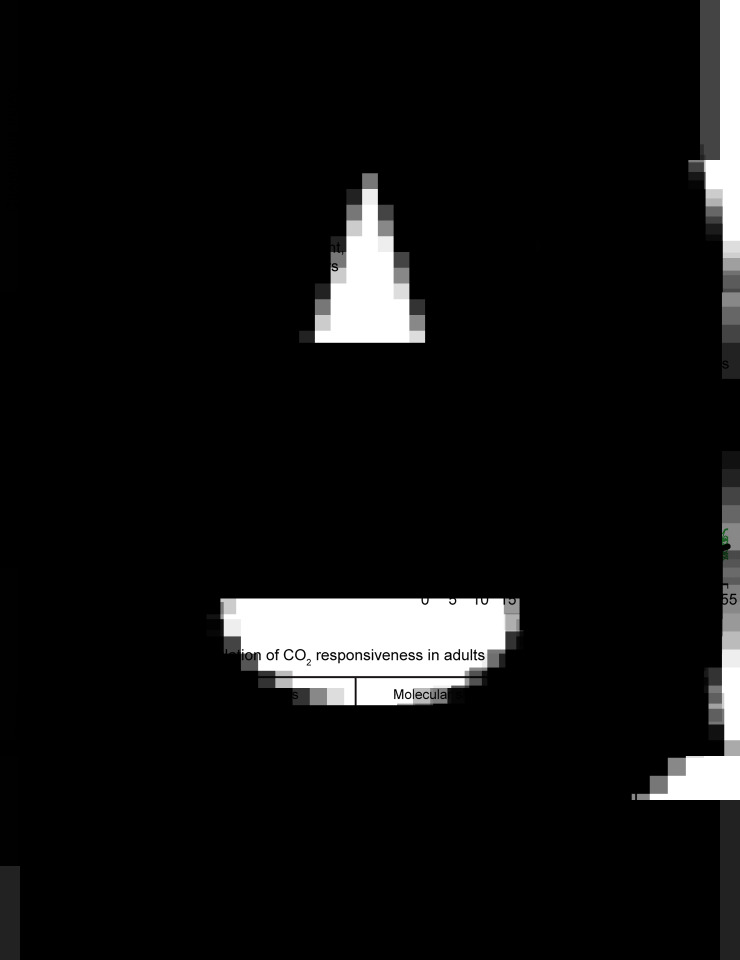
Behavioural responses of *C. elegans* to CO_2_. (A) Responses of wild-type *C. elegans* adults and dauers to CO_2_ in a chemotaxis assay (Fig. 1A). Dauers are developmentally arrested third-stage larvae that are similar to parasitic iL3s and IJs (Hotez *et al*., 1993; Viney *et al*., 2005; Crook, 2014). Animals were either well-fed adults cultivated at ambient CO_2_, well-fed adults cultivated at high CO_2_, starved adults cultivated at ambient CO_2_ or dauer larvae cultivated at ambient CO_2_. Adults were tested in a 20 min assay; dauer larvae were tested in a 1 h assay. Responses shown are to 2.5% CO_2_ (for adults cultivated at high CO_2_) or 10% CO_2_ (for all other conditions). For the high CO_2_ condition, adults were cultivated at 2.5% CO_2_ for one generation prior to the assay. For the starvation condition, adults were starved for 3 h prior to the assay. Data are from Guillermin *et al*. (2017), Rengarajan *et al*. (2019) and Hallem *et al*. (2011*a*). ****P < 0.0001, one-way ANOVA with Dunnett's post-test. The graph shows medians and interquartile ranges. (B) Previously experienced hypoxic conditions modulate CO_2_ responsiveness in *C. elegans* adults. Animals cultivated at low (1%) oxygen for 1 h prior to assays showed decreased CO_2_ avoidance. This change is mediated by hypoxia inducible factor-1 (HIF-1), since *hif-1* mutants are not affected by prior O_2_ exposure. ns, not significant relative to N2 (wild-type); ***P < 0.001 relative to N2; +++*P* < 0.001 relative to N2 exposed to 1% O_2_ for 1 h. The N2 (air) condition represents a control condition in which animals were not exposed to a CO_2_ gradient. The graph shows means and SEMs. Figure is from Bretscher *et al*. (2008), copyright 2008 National Academy of Sciences. (C) The BAG sensory neurons and GCY-9 are required for CO_2_ repulsion in *C. elegans*. BAG-ablated animals and *gcy-9* loss-of-function (*lf*) mutants are neutral to CO_2_. ****P < 0.0001, one-way ANOVA with Dunnett's post-test. The graph shows medians and interquartile ranges. Data are from Carrillo *et al*. (2013). (D) Calcium activity in BAG neurons in response to 10% CO_2_, as measured using the ratiometric calcium indicator yellow cameleon 3.60 (YC3.60). Green traces show responses of individual neurons; black line shows median response. Data are from Rengarajan *et al*. (2019). (E) A model for experience-dependent modulation of CO_2_ responsiveness in *C. elegans* adults. Under ambient conditions, CO_2_ repulsion is mediated by activation of the RIA and RIG interneurons and inhibition of the AIY interneurons, and by both dopamine and neuropeptide signalling involving the neuropeptide gene *nlp-1*. CO_2_ attraction in animals cultivated at high CO_2_ is mediated by activation of AIY, inhibition of RIA and silencing of RIG, as well as neuropeptide signalling involving the neuropeptide gene *flp-16*. Finally, CO_2_ attraction in starved adults is mediated by silencing of RIG and by a change in AIY responses such that activation and inhibition are observed with approximately equal frequency. Octopamine signalling and neuropeptide signalling *via* the neuropeptide genes *nlp-1* and *flp-16* also promote CO_2_ attraction during starvation. Blue = excitatory activity, orange = inhibitory activity, grey = silencing of activity. Figure is adapted from Guillermin *et al*. (2017) and Rengarajan *et al*. (2019).

The first studies of CO_2_ responsiveness in *C. elegans* demonstrated that these worms undergo rapid changes in locomotion in response to changes in CO_2_ concentrations (Dusenbery, 1985). These responses are characterized by an overall decrease in movement and an increase in turning frequency (Dusenbery, 1985). A more recent study examining the effects of acute CO_2_ exposure found that freely moving well-fed adults reverse rapidly when their head is exposed to high levels of CO_2_, indicating that CO_2_ is an aversive cue for well-fed *C. elegans* adults (Hallem and Sternberg, 2008). In addition, well-fed *C. elegans* adults avoid high CO_2_ areas when allowed to migrate along a CO_2_ gradient in a CO_2_-chemotaxis assay (Fig. 3A) (Bretscher *et al*., 2008). For well-fed adults, CO_2_ may indicate the presence of potential predators or pathogens, and repulsion from CO_2_ may function as an escape mechanism.

As is the case for some parasitic nematodes, CO_2_ responsiveness in *C. elegans* is subject to experience-dependent plasticity. In the case of *C. elegans*, one of the factors that influences CO_2_ responsiveness is the worm's nutritional status. Depriving adults of food suppresses CO_2_ avoidance behaviour (Bretscher *et al*., 2008; Hallem and Sternberg, 2008). Moreover, as *C. elegans* adults are starved, CO_2_ response shifts from repulsion to attraction (Fig. 3A) (Rengarajan *et al*., 2019). The effects of starvation can be reversed when the animals are re-exposed to food (Rengarajan *et al*., 2019). The shift from CO_2_ repulsion to CO_2_ attraction in starved worms may be beneficial for survival, since starved animals must find food in order to survive and bacterial food emits CO_2_. CO_2_ attraction by starved animals may also reflect an increased tolerance for risk taking; CO_2_ attraction is an inherently risky behaviour, since both predators and pathogens of *C. elegans* emit CO_2_ (Felix and Duveau, 2012; Brandt and Ringstad, 2015; Schulenburg and Felix, 2017).

CO_2_ responsiveness in *C. elegans* adults is also modulated by recently experienced environmental CO_2_ and oxygen (O_2_) levels, as well as immediate O_2_ context. For example, animals exposed to elevated CO_2_ levels (2.5% CO_2_) become robustly attracted to CO_2_ over the course of hours in a reversible manner (Fig. 3A) (Guillermin *et al*., 2017). Prior exposure to low O_2_ levels also suppresses CO_2_ avoidance in adults, an effect that depends on the hypoxia inducible factor gene *hif-1* (Fig. 3B) (Bretscher *et al*., 2008). In addition, whether animals have been pre-exposed to low O_2_ affects their responsiveness to CO_2_ stimuli under some conditions (Fenk and de Bono, 2017). CO_2_ responsiveness is also modulated by ambient O_2_ levels such that animals assayed under low O_2_ conditions are more strongly repelled by CO_2_ than animals assayed under high O_2_ conditions (Carrillo *et al*., 2013; Kodama-Namba *et al*., 2013). *C. elegans* prefers O_2_ concentrations below atmospheric (Gray *et al*., 2004); however, when exposed to opposing gradients of O_2_ and CO_2_, the avoidance response to high CO_2_ dominates over the avoidance response to high O_2_ (Bretscher *et al*., 2008). In nature, *C. elegans* is found in rotting vegetation (Felix and Braendle, 2010; Schulenburg and Felix, 2017), where both O_2_ and CO_2_ concentrations fluctuate. Moreover, both O_2_- and CO_2_-sensing pathways control foraging behaviour (Bendesky *et al*., 2011; Milward *et al*., 2011; Juozaityte *et al*., 2017). Thus, the interplay between O_2_- and CO_2_-evoked behaviours likely contributes to the ability of *C. elegans* to navigate the complex organic environments it inhabits.

CO_2_ responsiveness is also modulated by the presence or absence of food, and prior temperature experience (Bretscher *et al*., 2008, 2011; Kodama-Namba *et al*., 2013). In the case of temperature-dependent modulation of CO_2_ responsiveness, animals cultivated at 22 °C show enhanced repulsion to 1% CO_2_ when assayed at 15 °C compared with 22 °C (Kodama-Namba *et al*., 2013), suggesting an interaction between recent temperature experience and CO_2_ sensing. The ecological significance of this interaction is not yet clear, but it suggests that CO_2_ preferences may vary on a diurnal cycle as the ambient temperature fluctuates. Thus, CO_2_-evoked behaviours are regulated by multiple sensory modalities, resulting in both context-dependent and experience-dependent responses to CO_2_.

CO_2_ can also elicit behavioural changes in *C. elegans* that are independent of changes in locomotion. For example, exposing *C. elegans* to high CO_2_ levels (5% CO_2_) inhibits egg-laying behaviour, at least transiently (Fenk and de Bono, 2015). Modulation of egg-laying behaviour by high CO_2_ levels may prevent animals from exposing their progeny to unfamiliar environmental conditions. Feeding behaviour is also altered by CO_2_ such that brief exposure to high CO_2_ levels causes an acute reduction in pharyngeal pumping (Sharabi *et al*., 2009). Well-fed animals stop pharyngeal pumping when exposed to a lower concentration of CO_2_ than starved animals, suggesting that this behavioural change is dependent on the nutritional state of the animal (Sharabi *et al*., 2009).

Finally, *C. elegans* exhibits life-stage-specific responses to CO_2_. *C. elegans* dauers, which are developmentally arrested third-stage larvae that are similar to the iL3 and IJ stages of parasitic nematodes (Hotez *et al*., 1993; Viney *et al*., 2005; Crook, 2014), are attracted to CO_2_ (Fig. 3A) (Hallem *et al*., 2011*a*). Dauer larvae form when food is limited or environmental conditions are otherwise unfavourable (Hu, 2007), and CO_2_ attraction by dauers may enable them to locate bacterial food sources. In addition, dauers associate with invertebrates such as slugs, snails and isopods, which act as carriers that facilitate their dispersal to new niches (Baird, 1999; Caswell-Chen *et al*., 2005; Lee *et al*., 2012; Schulenburg and Felix, 2017). Thus, CO_2_ attraction may also enable dauers to locate and associate with invertebrate carriers.

### Effects of CO_2_ on *C. elegans* development and physiology

High levels of CO_2_ can also elicit physiological changes in *C. elegans*. Cultivating animals at CO_2_ levels above 9% CO_2_ decreases brood size and significantly slows the rate of embryonic development (Sharabi *et al*., 2009). This developmental delay is not caused by any obvious reduction in the overall health of the embryos or the adults that develop from these embryos. In addition, chronic exposure (>4 days) to 19% CO_2_ causes defects in overall body muscle morphology, resulting in long-lasting movement defects (Sharabi *et al*., 2009). Nevertheless, cultivation at 19% CO_2_ extends lifespan, an effect that appears to be independent of the inhibitory effect of CO_2_ on egg laying (Sharabi *et al*., 2009). The lifespan extension induced by exposure to high CO_2_ also may be independent of the dietary restriction pathway, since *eat-2* mutants, which show reduced pharyngeal pumping, show increased lifespan at high CO_2_ (Sharabi *et al*., 2009). Thus, CO_2_ has diverse effects on *C. elegans* development, physiology and behaviour.

### Behavioural and physiological responses of other free-living nematodes to CO_2_

Other free-living nematodes show diverse responses to CO_2_. For example, the free-living marine nematode *Adoncholaimus thalassophygas* is attracted to CO_2_ (Riemann and Schrage, 1988). This effect is not due to a general decrease in the pH of the medium, since the addition of hydrochloric acid did not elicit a similar attractive response (Riemann and Schrage, 1988). CO_2_ is emitted from bacteria present in decaying carcasses found in sediments and may act as a food signal for these nematodes. The necromenic nematode *Pristionchus pacificus*, which represents an evolutionary intermediate between free-living and parasitic nematodes, displays acute CO_2_ avoidance (Hallem and Sternberg, 2008). In contrast, the free-living nematodes *Caenorhabditis briggsae*, *Caenorhabditis angaria* and *Panagrellus redivivus* do not respond to CO_2_ in acute avoidance assays (Hallem and Sternberg, 2008). However, ambient CO_2_ concentrations play a prominent role in regulating reproduction in *Panagrellus redivivus.* Under low O_2_ conditions, the brood size of *Panagrellus redivivus* increases in response to an increase in CO_2_ concentration from 0 to 5% (Hansen and Buecher, 1970). The effects of CO_2_ on nematode physiology also vary greatly across species. At the extreme, nematodes have been isolated from volcanic gas vents, where CO_2_ levels can reach 100%; these nematodes can survive under 100% CO_2_ conditions for at least 5 days (Pilz and Hohberg, 2015). Thus, CO_2_ has species-specific effects on nematode behaviour and physiology.

## Cellular and molecular mechanisms of CO_2_ responsiveness in nematodes

### Cellular mechanisms of CO_2_ responsiveness in *C. elegans*

The primary CO_2_-sensing neurons in *C. elegans* are the paired BAG neurons in the head. Ablation of the BAG neurons abolishes both CO_2_ avoidance in adults (Hallem and Sternberg, 2008) and CO_2_ attraction in dauers (Fig. 3C) (Hallem *et al*., 2011*a*). Exposing animals to varying concentrations of CO_2_ produces dose-dependent calcium activity in the BAG neurons *in vivo* (Fig. 3D) (Hallem *et al*., 2011*b*). In addition, isolated BAG neurons derived from *C. elegans* embryos respond to CO_2_ *in vitro*, suggesting that BAG neurons are intrinsically sensitive to CO_2_ (Smith *et al*., 2013). The calcium responses in isolated BAG neurons in culture are independent of carbonic anhydrase activity, indicating that these neurons can sense molecular CO_2_. Moreover, a majority of isolated BAG neurons do not respond to pH, although responses to low pH are detectable in some isolated BAG neurons (Smith *et al*., 2013). However, the role of BAG neurons is not limited to promoting CO_2_ responses. The BAG neurons also sense O_2_ (Zimmer *et al*., 2009). Furthermore, they play a role in establishing food odour preferences and in foraging behaviour (Harris *et al*., 2014; Juozaityte *et al*., 2017). In addition to BAG neurons, other sensory neurons including ASE, AFD, AWC, ASJ, ASK, ASH and ADL also exhibit CO_2_-evoked calcium activity and contribute to CO_2_ responsiveness (Bretscher *et al*., 2011; Fenk and de Bono, 2015). The interneurons AIY, RIG, RIA and AIZ act downstream of BAG neurons to mediate CO_2_-evoked behaviour (Kodama-Namba *et al*., 2013; Guillermin *et al*., 2017). The AIA interneurons also show CO_2_-evoked activity and are involved in CO_2_ responsiveness (Fenk and de Bono, 2015). In well-fed adults, CO_2_ repulsion is correlated with activation of RIA, RIG and AIZ, and inhibition of AIY (Fig. 3E) (Guillermin *et al*., 2017).

### Molecular mechanisms of CO_2_ responsiveness in *C. elegans*

The detection of CO_2_ by the BAG neurons requires the receptor-type guanylate cyclase GCY-9. Animals with loss-of-function mutations in *gcy-9* are insensitive to CO_2_ in behavioural assays (Fig. 3C) (Hallem *et al*., 2011*b*). Moreover, CO_2_-evoked calcium transients in the BAG neurons require GCY-9, and ectopic expression of GCY-9 confers CO_2_ sensitivity to other sensory neurons (Hallem *et al*., 2011*b*; Brandt *et al*., 2012; Carrillo *et al*., 2013). The expression of GCY-9 in the BAG neurons requires the E26 transformation-specific (ETS)-domain transcription factor ETS-5, and *ets-5* mutants fail to avoid CO_2_ (Guillermin *et al*., 2011; Brandt *et al*., 2012). Both ETS-5 and the SoxD transcription factor EGL-13 are also required more generally for normal differentiation of the BAG neurons (Guillermin *et al*., 2011; Brandt *et al*., 2012; Petersen *et al*., 2013). The Toll-like receptor TOL-1 is also required for normal BAG neuron differentiation, and *tol-1* mutants are defective in pathogen avoidance behaviour as a result (Brandt and Ringstad, 2015).

In addition to GCY-9, the cGMP signalling pathway that mediates CO_2_ detection consists of the cGMP-gated cation channel TAX-2/TAX-4 (Bretscher *et al*., 2008; Hallem and Sternberg, 2008). Glutamate signalling and neuropeptide signalling are also required for BAG-mediated responses to CO_2_ (Guillermin *et al*., 2017). BAG neurons are glutamatergic (Serrano-Saiz *et al*., 2013), and well-fed adults lacking the vesicular glutamate transporter EAT-4 show neutral responses to CO_2_ (Guillermin *et al*., 2017). BAG neurons also release neuropeptides, and well-fed adults lacking the BAG-expressed FMRFamide-like neuropeptide FLP-17 do not respond to CO_2_ (Guillermin *et al*., 2017). In addition, several other signalling molecules, including the calcineurin subunits TAX-6 and CNB-1, the regulator of G-protein signalling RGS-3 and the nuclear hormone receptor NHR-49 are known to regulate CO_2_ response (Hallem and Sternberg, 2008). The microRNA *mir-791* is also required for the normal CO_2_-evoked calcium activity of the BAG neurons (Drexel *et al*., 2016).

### Mechanisms underlying the context-dependent modulation of CO_2_ responsiveness by O_2_

The extent to which CO_2_ responsiveness is regulated by ambient O_2_ levels depends on the neuropeptide Y receptor NPR-1 (McGrath *et al*., 2009; Carrillo *et al*., 2013; Kodama-Namba *et al*., 2013). The laboratory wild-type (N2) strain of *C. elegans* contains a gain-of-function mutation in the *npr-1* gene that confers CO_2_ avoidance on well-fed adults regardless of ambient O_2_ levels. However, animals containing loss-of-function (*lf*) mutations in *npr-1* and animals carrying the natural variant of *npr-1* avoid CO_2_ under low O_2_ conditions but do not respond to CO_2_ at normal atmospheric O_2_ levels (21% O_2_) (Carrillo *et al*., 2013; Kodama-Namba *et al*., 2013). The gain-of-function NPR-1 variant in N2 animals suppresses the activity of the O_2_-sensing URX neurons to promote CO_2_ avoidance regardless of ambient O_2_ levels. In animals containing an *npr-1(lf)* mutation or a natural variant of *npr-1*, the URX neurons are tonically active under high O_2_ conditions and inhibit CO_2_ avoidance at high O_2_. The RIA interneurons appear to act downstream of URX to partially mediate its effects on the CO_2_ circuit (Kodama-Namba *et al*., 2013). In addition, the neuroglobin gene *glb-5* also acts *via* the URX neurons to modulate CO_2_ responsiveness as a function of ambient O_2_ levels (McGrath *et al*., 2009; Kodama-Namba *et al*., 2013).

### Mechanisms underlying the experience-dependent modulation of CO_2_ responsiveness

The mechanisms underlying experience-dependent modulation of CO_2_ responsiveness in *C. elegans* have been elucidated in some detail. The shift in CO_2_ response from repulsion to attraction when animals are moved from low CO_2_ to high CO_2_ cultivation conditions results from the differential activity of a single set of interneurons downstream of the BAG sensory neurons (Guillermin *et al*., 2017). In animals that were previously cultivated at low CO_2_, CO_2_ exposure inhibits the AIY interneurons and activates the RIA and RIG interneurons. In contrast, in animals that have been cultivated at high CO_2_, CO_2_ exposure activates AIY and inhibits RIA. Moreover, RIG is silenced such that it no longer responds to CO_2_ (Fig. 3E). Thus, CO_2_ response is not determined by whether an ‘attractive’ or ‘repulsive’ pathway is activated; rather, it is determined by experience-dependent modulation of interneuron activity in a single pathway (Guillermin *et al*., 2017). A number of neuropeptides also differentially modulate CO_2_ responsiveness in animals cultured under high *vs* low CO_2_ conditions (Fig. 3E) (Guillermin *et al*., 2017).

The shift from CO_2_ repulsion to CO_2_ attraction that occurs during starvation also arises due to the differential activities of the AIY and RIG interneurons (Rengarajan *et al*., 2019). In starved animals, RIG is silenced and AIY shows stochastic responses such that CO_2_ evokes activating and inhibiting responses with approximately equal frequency (Fig. 3E). At the molecular level, whether CO_2_ is attractive or repulsive is regulated by biogenic amine signalling. Dopamine promotes CO_2_ avoidance in well-fed animals by promoting activation of RIG and inhibition of AIY, while octopamine promotes CO_2_ attraction in starved animals by promoting activation of AIY (Fig. 3E) (Rengarajan *et al*., 2019). Thus, the CO_2_ circuit is modulated during starvation by opposing biogenic amine signals. Neuropeptide signalling also regulates CO_2_ responsiveness during starvation (Fig. 3E) (Rengarajan *et al*., 2019). Finally, CO_2_ attraction in dauer larvae is less well understood but is regulated at least in part by neuropeptide signalling (Lee *et al*., 2017).

### Molecular and cellular mechanisms underlying other CO_2_-evoked behaviours

Some of the molecular and cellular mechanisms that mediate the effects of CO_2_ on other behaviours in *C. elegans* have also been elucidated. For instance, CO_2_-evoked activity in the AWC sensory neurons triggers a cGMP signalling pathway that ultimately inhibits the activity of the HSN neurons, resulting in the inhibition of egg-laying behaviour (Fenk and de Bono, 2015). Antagonistic effects of the BAG neurons and the URX neurons regulate lifespan in *C. elegans*, resulting in increased longevity in BAG-ablated animals (Liu and Cai, 2013). Mutations in the c-Jun N-terminal kinase (JNK) signalling pathway genes *jnk-1* and *kgb-2* suppress CO_2_-induced fertility defects, indicating that JNK signalling may be involved in regulating fertility in response to CO_2_ (Vadasz *et al*., 2012).

### Unanswered questions regarding CO_2_ responsiveness in *C. elegans*

Although the mechanisms underlying CO_2_ responsiveness in *C. elegans* have been elucidated in appreciable detail, several questions remain unexplored. For example, more information is needed to fully understand how the differential flow of information from BAG neurons to downstream interneurons generates experience-dependent plasticity of CO_2_-evoked behaviour. One intriguing possibility is that the BAG neurons express or release different neurotransmitters or neuropeptides in response to CO_2_ under varying conditions. Consistent with this possibility, the BAG neurons modulate the expression of FLP-19 neuropeptides as a function of their CO_2_-evoked activity (Rojo Romanos *et al*., 2017). In addition, the interneurons that act downstream of other CO_2_-sensing neurons have not been identified. Finally, the CO_2_ microcircuit that drives CO_2_ attraction in dauers remains poorly understood, although it appears to involve dauer-specific, gap-junction-mediated signalling between the BAG neurons and the downstream AIB interneurons (Bhattacharya *et al*., 2019). In future studies, it will be interesting to determine whether the same set of neurons or a distinct set of neurons promotes CO_2_ attraction in dauers. A better understanding of the neural circuits and signalling pathways that regulate CO_2_ responsiveness as a function of experience, context and life stage will provide important insights into how a single sensory cue can give rise to diverse behavioural responses in an ethologically-appropriate manner.

### Mechanisms underlying CO_2_ responsiveness in other nematodes

The anatomy and function of nematode sensory neurons are generally conserved across species (Ashton *et al*., 1995, 1999; Lopez *et al*., 2000; Li *et al*., 2000*a*, 2000*b*, 2001; Bhopale *et al*., 2001; Forbes *et al*., 2004; Ketschek *et al*., 2004; Ashton *et al*., 2007; Bumbarger *et al*., 2007; Srinivasan *et al*., 2008; Bumbarger *et al*., 2009; Zhu *et al*., 2011; Hallem *et al*., 2011*a*), making it possible to use knowledge of CO_2_ responsiveness in *C. elegans* as a starting point for launching investigations into the mechanisms of CO_2_ responsiveness in parasitic nematodes. In the case of both the necromenic nematode *Pristionchus pacificus* and the EPNs *Heterorhabditis bacteriophora* and *Steinernema carpocapsae*, BAG neurons were identified on the basis of conserved neuroanatomical position and shown to be required for behavioural responses to CO_2_ by laser ablation analyses (Hallem and Sternberg, 2008; Hallem *et al*., 2011*a*). BAG-ablated *Pristionchus pacificus* adults do not show acute CO_2_ avoidance, and BAG-ablated *Heterorhabditis bacteriophora* and *Steinernema carpocapsae* IJs do not show CO_2_ attraction (Hallem *et al*., 2011*a*). In addition, CO_2_-evoked jumping behaviour in *Steinernema carpocapsae* requires the BAG neurons (Hallem *et al*., 2011*a*). Thus, the neural circuits that mediate CO_2_ response are at least partly conserved across nematode species. However, the interneurons that operate downstream of BAG neurons to mediate CO_2_ responsiveness in other nematode species have not yet been identified. Moreover, nothing is currently known about the neural circuits and molecular signals that promote CO_2_ responsiveness in mammalian-parasitic nematodes. In future studies, it will also be interesting to determine whether similar or distinct mechanisms operate in *C. elegans* and parasitic nematodes to modulate CO_2_ responses depending on context, previous experience or life stage.

## Directions for future research

A major focus going forward will be on elucidating the cellular and molecular mechanisms underlying CO_2_ responsiveness in mammalian-parasitic nematodes. The identification of the neural mechanisms that drive or regulate the CO_2_ responses of mammalian-parasitic nematodes both inside and outside the host could lead to the identification of new drug targets or new strategies for nematode control. Until recently, investigations into the mechanisms underlying sensory behaviours in parasitic nematodes were limited to laser ablation analysis due to the dearth of resources and tools required for the genetic manipulation of these parasites. Laser ablation analysis has been used to establish the function of a number of different sensory neurons in mammalian-parasitic nematodes, including *Strongyloides stercoralis*, hookworms and *Haemonchus contortus* (Ashton *et al*., 1998; Lopez *et al*., 2000; Li *et al*., 2000*b*; Bhopale *et al*., 2001; Forbes *et al*., 2004; Ketschek *et al*., 2004; Nolan *et al*., 2004; Ashton *et al*., 2007). However, several recent advances have facilitated the study of gene function and the genetic basis of sensory behaviours in these parasites. High-quality genome sequences of several nematode species have been identified and are readily accessible (Mitreva *et al*., 2007; Brindley *et al*., 2009; Hunt *et al*., 2016; Howe *et al*., 2017; International Helminth Genomes, 2019). Transcriptomic data are also now available for many parasitic nematode species (Jex *et al*., 2019), which may significantly advance the study of gene expression and the identification of novel signalling pathways that drive sensory behaviours.

The most genetically tractable parasitic nematodes are *Strongyloides stercoralis* and *Strongyloides ratti. Strongyloides stercoralis* and *Strongyloides ratti* are more readily amenable to genetic manipulation than other parasitic nematodes because they can undergo one free-living generation (Viney, 1999, 2006; Lok, 2007). Foreign DNA can be introduced by gonadal microinjection into free-living adults using techniques based on those originally developed for *C. elegans* (Evans, 2006). Most other mammalian-parasitic nematodes lack a free-living generation, which makes it difficult to introduce foreign DNA into these worms. *Strongyloides stercoralis* is a human parasite that infects approximately 370 million people worldwide (Page *et al*., 2018) and is therefore of direct interest as a human pathogen; additionally, *Strongyloides stercoralis* is of interest as a model for other human-parasitic nematodes such as hookworms that cannot be genetically manipulated.

Transgenic nematodes can be generated by introducing plasmid DNA containing exogenous genes; these genes are then expressed as extrachromosomal arrays in the F_1_ progeny of the microinjected adults (Lok and Massey, 2002; Li *et al*., 2006, 2011; Junio *et al*., 2008; Lok and Artis, 2008; Lok, 2012; Shao *et al*., 2012; Lok *et al*., 2017; Shao *et al*., 2017). This technique can be potentially used to express any gene of choice, including those required for genetic ablation or silencing of neurons (Schiavo *et al*., 1992; Qi *et al*., 2012; Williams *et al*., 2013; Pokala *et al*., 2014) and those required for monitoring neuronal activity (Nagai *et al*., 2004; Chen *et al*., 2013; Dana *et al*., 2016). The targeted expression of exogenous genes in *Strongyloides* has been aided by the identification of several promoters that drive expression in single cells or subsets of cells (Junio *et al*., 2008; Stoltzfus *et al*., 2012; Bryant *et al*., 2018). However, whereas extrachromosomal arrays in *C. elegans* are stably expressed across generations, extrachromosomal arrays in *Strongyloides* are silenced after the F_1_ generation by as-yet-unknown mechanisms (Junio *et al*., 2008; Li *et al*., 2011). Persistent expression across generations can be achieved in *Strongyloides* by methods that promote genomic integration of transgenes, such as transposon-mediated random integration (Shao *et al*., 2012; Lok, 2013) and CRISPR/Cas9-mediated targeted integration (Gang *et al*., 2017).

**Fig. 4.**
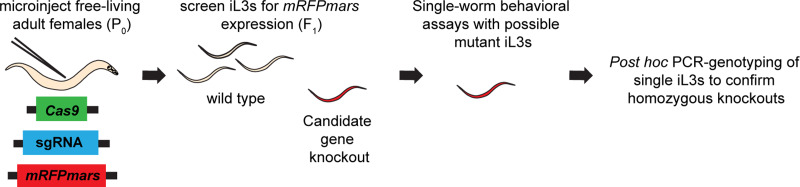
Targeted mutagenesis in *Strongyloides stercoralis.* (A) Strategy for CRISPR/Cas9-mediated targeted mutagenesis in *Strongyloides stercoralis*. Plasmid vectors encoding Cas9, the single guide RNA (sgRNA) for the gene of interest and a repair template for homology-directed repair encoding an mRFPmars reporter are introduced into *Strongyloides stercoralis* free-living adult females (*P*_0_) by gonadal microinjection. The iL3 progeny (*F*_1_) from microinjected females are screened for *mRFPmars* expression, indicative of a possible disruption of the gene of interest. iL3s are then tested in single-worm chemotaxis assays and genotyped *post hoc* for homozygous disruption of the gene of interest. Figure is adapted from Gang *et al*. (2017).

Methods for disrupting gene function are also now available for *Strongyloides stercoralis* and *Strongyloides ratti*. The recent development of an approach for CRISPR/Cas9-mediated targeted gene disruption in these species provided the first insights into the genetic mechanisms that drive sensory behaviours (Fig. 4) (Gang *et al*., 2017; Lok *et al*., 2017; Bryant *et al*., 2018). For example, knockout of the gene encoding the cyclic-nucleotide-gated channel subunit TAX-4 severely disrupts the thermosensory behaviour of *Strongyloides stercoralis* infective larvae (Bryant *et al*., 2018). RNA interference (RNAi) has also now been successfully applied to *Strongyloides ratti*. RNAi approaches using both dsRNA and siRNA have been used to study the effects of transcriptional knockdown of genes in several parasitic nematode species, although with varying efficacy (Geldhof *et al*., 2006; Kotze and Bagnall, 2006; Visser *et al*., 2006; Kang and Hong, 2008; Lendner *et al*., 2008; Viney and Thompson, 2008; Samarasinghe *et al*., 2011; Britton *et al*., 2012; Zawadzki *et al*., 2012; Tzelos, 2014). In the case of *Strongyloides ratti*, a recent study demonstrated the first successful knockdown of multiple mRNAs using an siRNA approach (Dulovic and Streit, 2019). In addition, chemical mutagenesis has been used to perform unbiased forward genetic screens to generate dominant non-targeted mutations in *Strongyloides ratti* iL3s, although mapping the locations of these mutations has not been possible yet (Viney *et al*., 2002; Guo *et al*., 2015).

Using a combination of the above approaches, it should be possible to identify the neural mechanisms and molecular pathways that are involved in driving behavioural and physiological responses of *Strongyloides stercoralis* to CO_2_. For example, it will be interesting to determine whether the BAG neurons, which sense CO_2_ and promote behavioural responses to CO_2_ in *C. elegans*, play a similar role in *Strongyloides stercoralis.* It will also be important to elucidate the neural circuitry that operates downstream of the CO_2_-sensing neurons to mediate or modulate CO_2_-evoked behaviours in *Strongyloides stercoralis*. An intriguing possibility is that while sensory neuron function may be generally conserved across species, interneuron function may be less well conserved and may instead reflect species-specific behavioural and physiological responses to CO_2_. In addition, through the systematic screening of candidate genes known to be involved in CO_2_ responsiveness in *C. elegans*, it might be possible to uncover molecular signals that regulate parasite–host interactions or that are required for successful parasitism. In the long run, a better understanding of the molecular and cellular bases of CO_2_-evoked behaviours in parasitic nematodes may lead to new avenues for nematode control. It may also shed light on some of the unique sensory mechanisms that operate in parasitic nematodes to shape parasite-specific behavioural responses.
